# Prevalence of Professional Interpreter Use in Emergency Departments: A Systematic Review and Meta‐Analysis

**DOI:** 10.1111/acem.70427

**Published:** 2026-10-14

**Authors:** Henry Li, Ana Ramirez, Lisa Hartling, Jaspreet Khangura, Liz Dennett, Victor Do, Kayathiri Ganeshamoorthy, Samina Ali

**Affiliations:** ^1^ Department of Emergency Medicine, Faculty of Medicine and Dentistry University of Alberta Edmonton Alberta Canada; ^2^ Department of Pediatrics, Faculty of Medicine and Dentistry University of Alberta Edmonton Alberta Canada; ^3^ MD Program, Faculty of Medicine and Dentistry University of Alberta Edmonton Alberta Canada; ^4^ Women and Children's Health Research Institute, Faculty of Medicine and Dentistry University of Alberta Edmonton Alberta Canada; ^5^ Alberta Research Centre for Health Evidence, Department of Pediatrics, Faculty of Medicine and Dentistry University of Alberta Edmonton Alberta Canada; ^6^ Geoffrey and Robyn Sperber Health Sciences Library University of Alberta Edmonton Alberta Canada; ^7^ Provincial Interpretation and Translation Services Alberta Health Services Edmonton Alberta Canada

**Keywords:** accident and emergency, health equity, language barriers, language interpretation, language translation, meta‐analysis, prevalence, systematic review

## Abstract

**Background:**

Emergency departments (EDs) increasingly serve linguistically diverse populations. Professional interpretation improves care, yet its use remains variable. Quantifying the prevalence of use and understanding disparities can inform policies and interventions. We conducted a systematic review and meta‐analysis to estimate the prevalence of professional interpreter use in English‐speaking EDs and compare use across patient populations and countries.

**Methods:**

Eight databases were searched from inception to May 15, 2025. We screened full texts included in a prior scoping review to identify studies reporting the prevalence of professional interpreter use among patients requiring interpretation in English‐speaking EDs. We pooled prevalence overall and stratified by population (adult vs. children) and country (United States vs. other), with 95% prediction intervals and confidence intervals (CIs). Meta‐regression assessed associations between study year and prevalence. Risk of bias was evaluated using the JBI checklist for prevalence studies.

**Results:**

Thirty‐two studies spanning 1997–2025 involving 65,736 participants were included. Professional interpreters were used in 29% (CI, 20%, 38%) of encounters with patients requiring interpretation. Use was higher with children (49%; CI, 41%, 58%) than adults (16%; CI, 11%, 22%), and higher in the United States (33%; CI, 24%, 43%) than other countries (14%; CI, 4%, 30%). There was no significant association between study year and interpreter use (*p* = 0.38).

**Conclusions:**

Despite known benefits, professional interpreter use in EDs remains low, particularly among adults and outside the United States. Addressing this gap will require coordinated efforts from clinicians, health system leaders, and policymakers to improve access to and accountability for language‐concordant care in EDs.

## 1 Introduction

Emergency departments (EDs) in English‐speaking countries increasingly serve linguistically diverse populations. In the United States (U.S.), approximately 8% of individuals have limited English proficiency, and in Canada, 12% primarily speak a non‐official language at home [1, 2]. Patients with preferred languages other than English often encounter communication barriers in the ED, which are associated with increased diagnostic testing [3, 4, 5, 6, 7] and healthcare utilization [3, 4] as well as worse patient satisfaction [8] compared to English‐preferring patients. Language barriers are also associated with decreased understanding of discharge instructions [9], contributing to higher rates of ED return visits and hospital readmissions [10, 11].

Professional medical interpretation improves patient satisfaction, communication, and health outcomes [12, 13, 14]. Despite these benefits, use of professional interpreters in the ED appears highly variable, with reported rates ranging from 0.3% in one Canadian ED [15] to 45.4% in an American pediatric ED [16]. However, the overall prevalence of professional interpreter use in English‐speaking EDs—and how it differs across patient populations and countries—has not been systematically quantified. A comprehensive synthesis is needed to define the magnitude of this gap in evidence‐based communication support and to inform policy and quality improvement efforts.

Our objective was to estimate the global reported prevalence of professional interpreter use in English‐speaking EDs. We further examined differences in interpreter use across patient populations (encounters with children versus adults), countries, and time.

## 2 Methods

### 2.1 Study Design and Registration

We conducted a systematic review and meta‐analysis and report our results in accordance with the Preferred Reporting Items for Systematic Reviews and Meta‐Analyses (PRISMA) guidelines (Table S1) [17]. The protocol was registered with PROSPERO (CRD420251101780) prior to data extraction. Where possible, we followed recommendations from the Prevalence Estimates Reviews–Systematic Review Methodology Group (PERSyst) [18]. This includes the addition of prediction intervals [19], which is a change from our registered protocol. Research ethics approval was not required for this systematic review.

This systematic review builds on a previous scoping review on interpretation and translation in emergency care [20]. The search strategy and screening process were completed as a part of this scoping review using a broader set of inclusion criteria; we included any original study on interpretation and translation in emergency care settings (ED, urgent care, pre‐hospital; full inclusion criteria in Table S2). Studies included in the preceding scoping review were re‐screened for adherence to a narrower set of inclusion criteria for this systematic review and meta‐analysis (see below).

### 2.2 Search and Screening

A health sciences librarian (LD) developed the search strategy for the preceding scoping review (Supporting Information Methods) in consultation with content and methods experts. The strategy was peer reviewed using the Peer Review of Electronic Search Strategies (PRESS) checklist [21]. We searched MEDLINE, Embase, PsycINFO, CINAHL, Scopus, iPortal, Native Health Database, and Cochrane CENTRAL from database inception to May 20, 2025, without restrictions on date, country, or language. Search terms included subject headings and keywords related to language interpretation services and emergency care. For the emergency care concept, we used the filter by Campbell and Dennett [22]. Opinion pieces, letters, editorials, and news articles were removed when immediately identifiable. Search results were uploaded to Covidence and de‐duplication was performed. Two independent reviewers screened titles and abstracts, followed by full texts to identify studies to include in the preceding scoping review (inclusion criteria in Table S2) [20]. References and citations of all included studies were searched for additional studies. Disagreements at all stages were resolved through consensus between the two reviewers, and if consensus could not be reached, by a third reviewer.

The full texts of the studies included in the scoping review were then re‐screened by two independent reviewers for adherence to the narrower inclusion criteria of this systematic review (described below). The screening process was pilot‐tested with 10 full texts, then reviewers discussed interpretation of inclusion criteria to ensure they were clear and applied consistently before proceeding with the remaining articles. Disagreements were resolved through consensus. Any relevant articles/studies encountered during our work that were not captured by the systematic search were also considered and included if they met the inclusion criteria, following a snowballing approach.

### 2.3 Inclusion Criteria

In this systematic review, we included peer‐reviewed primary research studies that:

1. Were conducted in English‐speaking EDs;
2. Included patients of any age (or caregivers of children) requiring interpretation between English and another language; and
3. Reported the prevalence of professional interpreter use as a proportion or percentage among patients requiring interpretation.

We included before‐after and interrupted time series studies, but excluded interventional studies in which interpretation was allocated to individual patients (e.g., randomized trials). We also excluded studies reporting only ad hoc interpretation (e.g., family members or untrained staff), and studies looking at language translation (i.e., translation of written text). Interpretation was defined as “the rendering of live utterances in one language to live utterances in another language” which includes both spoken and signed languages [23]. Common professional interpretation modalities include in‐person, audiovisual, and telephonic.

We use the term preferred languages other than English to reflect a strengths‐based framing of linguistic diversity. However, we recognize that patients with language needs are identified in the literature using other descriptors such as limited English proficiency, request for an interpreter, native/first language other than English, and language most often spoken at home.

### 2.4 Data Extraction

After pilot‐testing with five full‐text articles to discuss interpretation of variables and approaches to extraction, two reviewers independently extracted data using a structured data extraction form (Table S3). Extracted variables included study characteristics, setting, participant demographics, electronic medical record use, interpretation modality, and prevalence of professional interpreter use. Disagreements were resolved by consensus.

For repeated cross‐sectional studies, we extracted the most recent estimate. For before‐after or interrupted time series studies involving an intervention, we extracted the most recent pre‐intervention estimate. When the prevalence was reported as a percentage without a numerator or denominator, we contacted the study's corresponding author via email to obtain the missing data.

### 2.5 Risk of Bias Assessment

Two reviewers independently assessed risk of bias using the Joanna Briggs Institute's (JBI) checklist for prevalence studies, which is the recommended tool for critical appraisal by the PERSyst methodological consortium [24, 25]. Studies were categorized as low (≥ 70% of criteria present), moderate (50%–69% of criteria present), or high risk of bias (< 50% of criteria present) as done in prior studies [26, 27]. We defined an adequate sample size as a minimum of 369 participants using the JBI prevalence sample size calculations and an expected prevalence of 40% based on prior studies, 95% confidence, and ±5% precision. Disagreements were resolved by consensus.

### 2.6 Data Synthesis and Meta‐Analysis

The primary outcome was the reported prevalence of professional interpreter use among patients with language needs (any definition, as described above). Prevalence was calculated as the reported number of eligible patients who received professional interpretation divided by the total number of eligible patients with language needs and expressed as a percentage. Patients with missing data were excluded because it was not possible to determine whether or not they received interpretation. Patients who were described to have declined interpretation or were seen by bilingual clinicians were excluded from the denominator, as non‐use reflected patient preference or language concordance. Studies that reported prevalence only by individual stages of care (e.g., triage, registration, physician assessment) without providing an overall per‐patient prevalence estimate were excluded from the meta‐analysis and summarized descriptively.

Pooled prevalence estimates were calculated using random‐effects meta‐analysis with double arcsine transformation [28], with stratified meta‐analyses by country (U.S. vs. other), population (adult vs. children), and risk of bias (low, moderate, high). We used the double arcsine transformation because it remains a widely used approach for meta‐analysis of prevalence estimates and accommodates studies with extreme proportions without requiring continuity corrections. Pooled estimates, 95% confidence intervals (which denote confidence in the current estimates), and prediction intervals (which predict the range of future potential values) [19, 29] were back‐transformed to the original proportion scale for presentation using the default inverse transformation implemented in the meta package.

Because concerns have been raised regarding back‐transformation of pooled estimates under certain circumstances [30], we performed a sensitivity analysis using generalized linear mixed models with a binomial likelihood and logit link as well as Clopper‐Pearson confidence intervals. Between‐study heterogeneity was quantified using *I* ^2^, which we interpret cautiously given the expected variability in prevalence estimates [19]. We conducted a univariable meta‐regression analysis to assess for associations between study year (i.e., when the study was conducted) and professional interpreter use. Evidence of publication bias was examined using a funnel plot and the Egger test. Statistical significance was defined as a two‐sided *p* < 0.05. All analyses were performed using R Studio (version 2025.09.2, build 26200) and R (version 4.5.2).

## 3 Results

### 3.1 Study Characteristics

Of 2101 search results and 95 studies identified in the related scoping review, 32 studies spanning 1997–2025 involving 65,736 participants (range 9–37,500, median 133.5) were included in this systematic review (Figure 1, Table 1) [15, 16, 31, 32, 33, 34, 35, 36, 37, 38, 39, 40, 41, 42, 43, 44, 45, 46, 47, 48, 49, 50, 51, 52, 53, 54, 55, 56, 57, 58, 59, 60]. For full‐text screening, the inter‐rater agreement was 86% and Cohen's kappa was 0.71. Individual study characteristics are presented in Table S4. Most studies were single‐center (*n* = 30, 94%), conducted after 2009 (*n* = 24, 75%), completed in the U.S. (*n* = 26, 81%), and at academic centers (*n* = 24, 75%). Non‐U.S. countries included Canada (*n* = 1), Australia (*n* = 4), and the United Kingdom (UK) (*n* = 1). One third of studies (*n* = 11, 34%) were conducted in pediatric‐only EDs, and three studies (9.4%) were conducted in adult‐only EDs. The most common modality available was telephonic interpretation (*n* = 19, 59%) followed by in‐person (*n* = 14, 44%) and audiovisual (*n* = 10, 31%). Eleven studies (34%) which were all U.S.‐based included only Spanish‐speaking patients, and the remaining studies included multiple languages.

**FIGURE 1:**
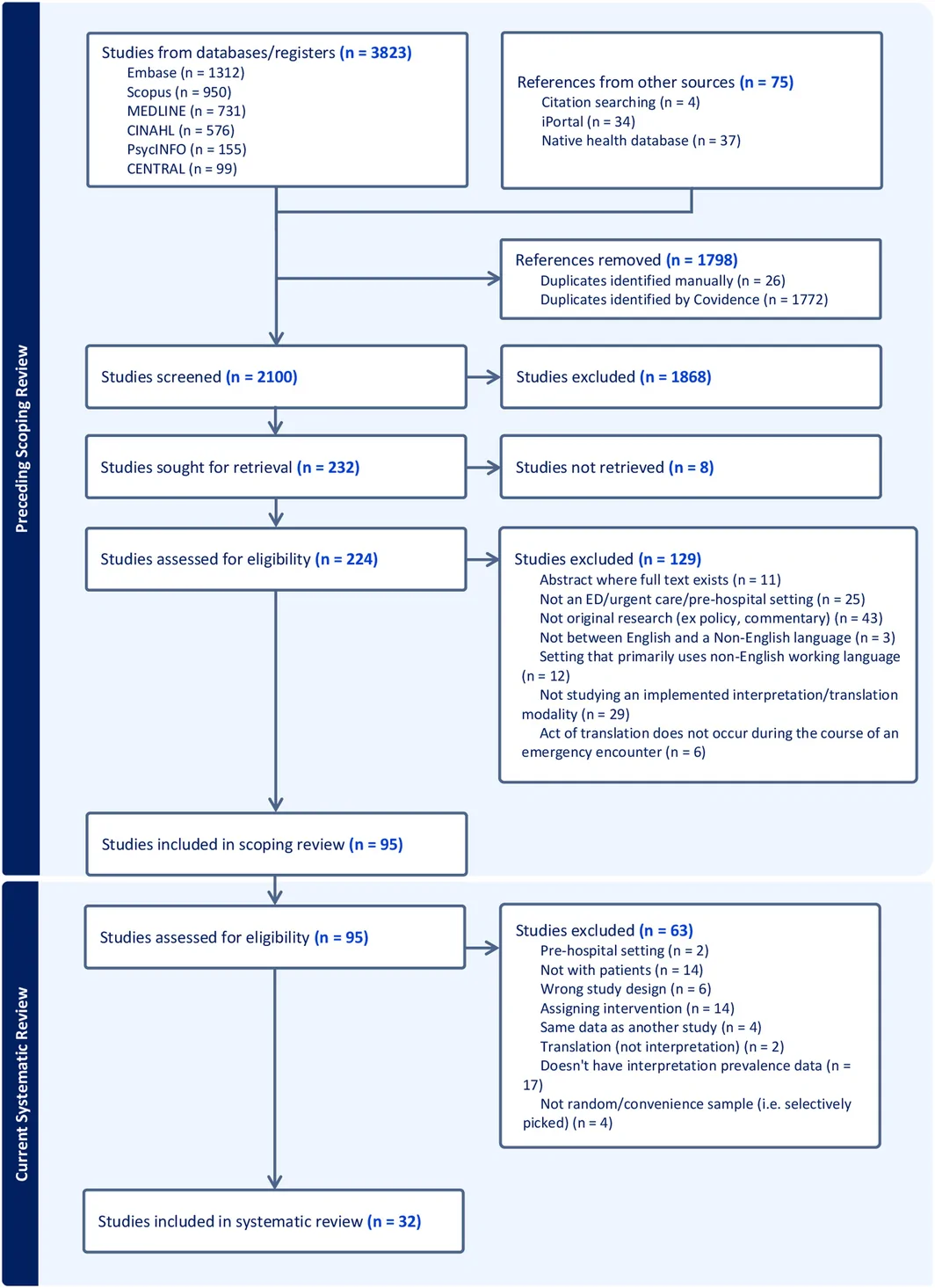
PRISMA flow diagram.

**TABLE 1:** Summary of study characteristics.

Summary of study characteristics		Number of studies (*N* = 32)
Publication type, *N* (%)	Abstract	6 (18.8%)
	Full text	26 (81.3%)
Decades, *N* (%)	1990s	4 (12.5%)
	2000s	4 (12.5%)
	2010s	19 (59.4%)
	2020s	5 (15.6%)
Country, *N* (%)	United States	26 (81.3%)
	Australia	4 (12.5%)
	Canada	1 (3.1%)
	United Kingdom	1 (3.1%)
Study design, *N* (%)	Cross‐sectional	12 (37.5%)
	Retrospective cohort	9 (28.1%)
	Prospective cohort	6 (18.8%)
	Before‐after (without comparison)	1 (3.1%)
	Interrupted time series (without comparison)	3 (9.4%)
	Interrupted time series (with comparison)	1 (3.1%)
Number of sites, *N* (%)	Single	30 (93.8%)
	Multicenter	2 (6.3%)
Setting, *N* (%)	General ED	6 (18.8%)
	Adult ED	3 (9.4%)
	Pediatric ED	11 (34.4%)
	Urgent Care	2 (6.3%)
	ED not described	11 (34.4%)
Academic center, *N* (%)	Yes	24 (75.0%)
	No	1 (3.1%)
	Not specified	7 (21.9%)
Interpreter modality, *N* (%)	In‐person	14 (43.8%)
	Audiovisual	10 (31.3%)
	Telephonic	19 (59.4%)
	Certified healthcare worker	2 (6.3%)
	Not specified	11 (34.4%)
Number of patients included, median (IQR)		133.5 (323.5)
Sample size range, *N* (%)	0–100	11 (34.4%)
	101–1000	16 (50.0%)
	> 1000	5 (15.6%)
Patient ages, *N* (%)	Adults	11 (34.4%)
	Children	12 (37.5%)
	Both	3 (9.4%)
	Not specified	6 (18.8%)

### 3.2 Risk of Bias

Risk of bias (Table 2) was low in seven studies (22%), moderate in 12 (38%), and high in 13 (41%). Most studies used an appropriate sample frame (*n* = 25, 78%), described their participants and settings in sufficient detail (*n* = 25, 78%), and used valid identifications of interpreter use (*n* = 28, 88%) which were measured in a standardized and reliable fashion (*n* = 30, 94%). No study reported confidence intervals for prevalence estimates, a requirement of the appropriate analysis criteria. Few studies had an adequate sample size (*n* = 10, 31%) or had sufficient coverage of their identified sample (*n* = 11, 34%), the latter primarily due to a lack of clarity on response rates by subgroups.

**TABLE 2:** Risk of bias assessments.

Study ID	D1	D2	D3	D4	D5	D6	D7	D8	D9	% met	Overall risk of bias
Leman (1997)	Yes	Yes	No	Yes	Unclear	Yes	Yes	No	No	56%	Moderate
Baker (1998)	Yes	No	No	Yes	Unclear	Yes	Yes	No	Yes	67%	Moderate
Bernstein (2002)	Yes	Yes	Yes	Yes	Yes	Yes	Yes	No	Yes	89%	Low
Hampers (2002)	No	Yes	Yes	Yes	Yes	Unclear	Yes	No	Yes	67%	Moderate
BonacruzKazzi (2003)	Yes	Yes	No	Yes	Yes	Yes	Yes	No	Yes	78%	Low
Garrett (2008)	Yes	No	No	Yes	Yes	Yes	Yes	No	Yes	67%	Moderate
Ginde (2010)	Yes	No	No	Yes	Yes	Yes	Yes	No	Yes	67%	Moderate
Flores (2012)	Yes	No	No	Yes	Unclear	Yes	Yes	No	Unclear	44%	High
Samuels‐Kalow (2013)	No	Yes	No	Yes	Unclear	Yes	Yes	No	No	44%	High
Mahmoud (2014)	No	No	No	Yes	Unclear	Yes	Yes	No	Unclear	33%	High
Wallbrecht (2014)	Yes	No	No	Yes	Yes	Yes	Yes	No	Yes	67%	Moderate
Armstrong (2015)	Yes	Unclear	No	No	Unclear	Yes	Yes	No	Unclear	33%	High
Arthur (2015)	Yes	Yes	No	Yes	Unclear	Yes	Yes	No	Yes	67%	Moderate
Cruz (2015)	Yes	No	No	No	Unclear	Yes	Yes	No	Unclear	33%	High
Ryan (2017)	Yes	Yes	Yes	Yes	Yes	Yes	Yes	No	Yes	89%	Low
Bitrus (2018)	Unclear	Unclear	No	No	Unclear	Yes	Yes	No	Unclear	22%	High
Benda (2019)	Yes	No	No	Yes	Unclear	Yes	Yes	No	Yes	56%	Moderate
Hartford (2019)	Yes	Yes	Yes	Yes	Yes	Yes	Yes	No	Yes	89%	Low
Marte (2019)	Yes	Yes	Yes	Yes	Unclear	Unclear	Unclear	No	Yes	56%	Moderate
Narang (2019)	Unclear	Unclear	No	No	Unclear	Yes	Yes	No	Yes	33%	High
Taira (2019)	Yes	No	No	Yes	Unclear	No	Yes	No	Yes	44%	High
Acharya (2020)	No	Unclear	No	No	Unclear	Yes	Yes	No	Yes	33%	High
Villalona (2020)	No	Yes	No	Yes	Unclear	Yes	Yes	No	Unclear	44%	High
Martinez (2021)	Yes	Yes	Yes	Yes	Unclear	Yes	Yes	No	Yes	78%	Low
Hartford (2022)	Yes	Yes	Yes	Yes	Yes	Yes	Yes	No	Yes	89%	Low
Jimal (2022)	Yes	Yes	Yes	Yes	Yes	Yes	Yes	No	Yes	89%	Low
Gupta (2023)	Yes	Yes	Yes	No	Unclear	Yes	Yes	No	Yes	67%	Moderate
Kundu (2023)	Yes	Yes	No	Yes	Unclear	Yes	Yes	No	Yes	67%	Moderate
Douglas (2024)	Yes	Yes	No	No	Unclear	Yes	Yes	No	No	44%	High
Hartford (2024)	Yes	No	No	Yes	No	Yes	Yes	No	No	44%	High
Torres (2024)	Yes	No	No	Yes	Unclear	Yes	Yes	No	Unclear	44%	High
Pavesi‐Krieger (2025)	Yes	Yes	Yes	Yes	Yes	No	Unclear	No	Yes	67%	Moderate

*Note:* Domain 1: Was the sample frame appropriate to address the target population? Domain 2: Were study participants sampled in an appropriate way? Domain 3: Was the sample size adequate? Domain 4: Were the study subjects and the setting described in detail? Domain 5: Was the data analysis conducted with sufficient coverage of the identified sample? Domain 6: Were valid methods used for the identification of the condition? Domain 7: Was the condition measured in a standard, reliable way for all participants? Domain 8: Was there appropriate statistical analysis? Domain 9: Was the response rate adequate, and if not, was the low response rate managed appropriately? Green is for Yes (for the domains) and low overall risk of bias. Yellow is for Unclear (for the domains) and moderate overall risk of bias. Red is for No (for the domains) and high overall risk of bias.

There was no evidence of publication bias on the funnel plot (Figure S1) or using Egger's test (*p* = 0.06).

### 3.3 Meta‐Analyses

Twenty‐eight studies were included in the meta‐analysis. The overall pooled prevalence of professional interpreter use was 29% (Figure 2, 95% CI, 20%, 38%) with substantial between‐study heterogeneity (*I* ^2^ = 99%). The 95% prediction interval ranged from 0% to 79%. In our sensitivity analysis using generalized linear mixed models, the overall pooled prevalence was 24% (95% CI, 16%, 35%).

**FIGURE 2:**
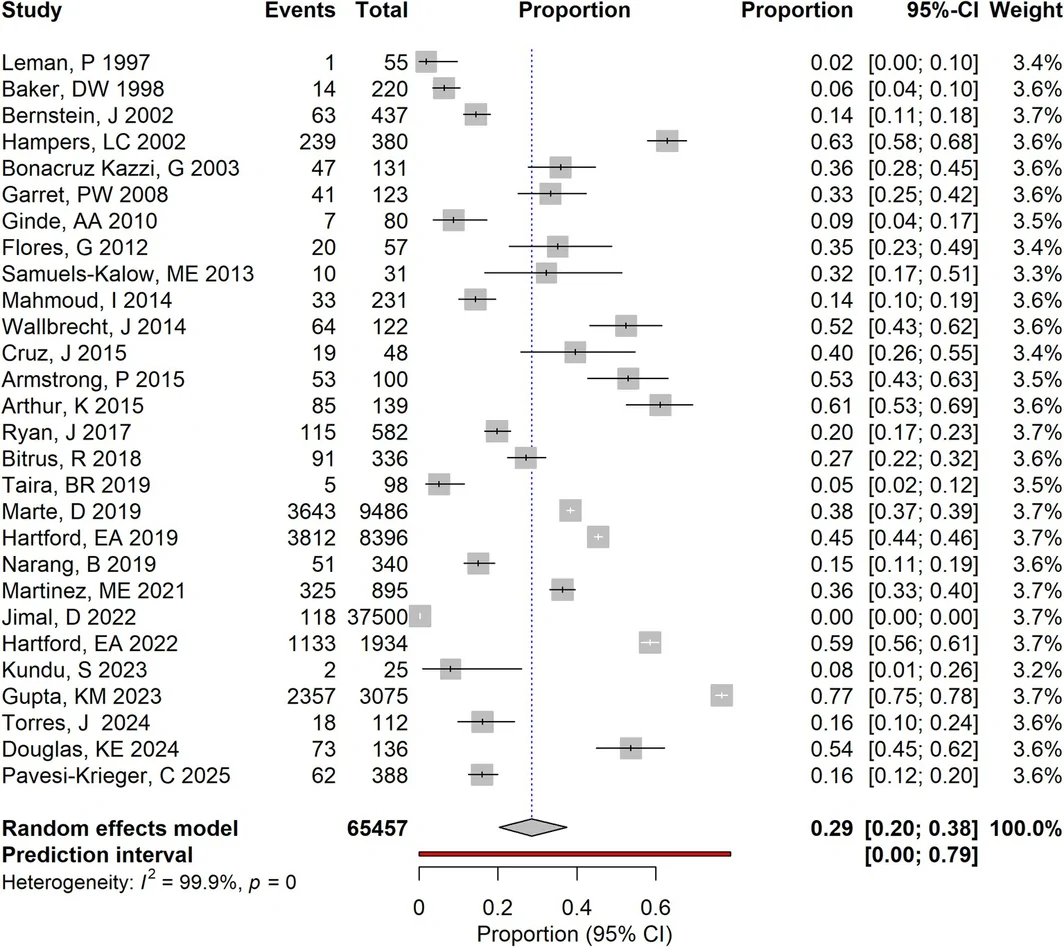
Overall pooled prevalence of professional interpretation.

In stratified analyses, pooled prevalence was higher in encounters with children (Figure 3, 49%; 95% CI 41%, 58%) [16, 45, 50, 51, 52, 53, 54, 55, 56, 57, 59] than in adults (16%; 95% CI, 11%, 22%) [31, 32, 34, 35, 36, 37, 38, 46, 47]. Prevalence of professional interpreter use was also higher in the U.S. (Figure 4, 33%; 95% CI, 24%, 43%) compared to other countries (14%; 95% CI, 4%, 30%). The latter group included a Canadian study with a prevalence of 0.3% [15], a study in the United Kingdom with a prevalence of 1.8% [48], and four Australian studies with prevalences ranging from 14% to 36% [31, 32, 45, 47]. There was no difference in pooled prevalence by risk of bias (Figure 5, low 27%, moderate 31%, high 27%).

**FIGURE 3:**
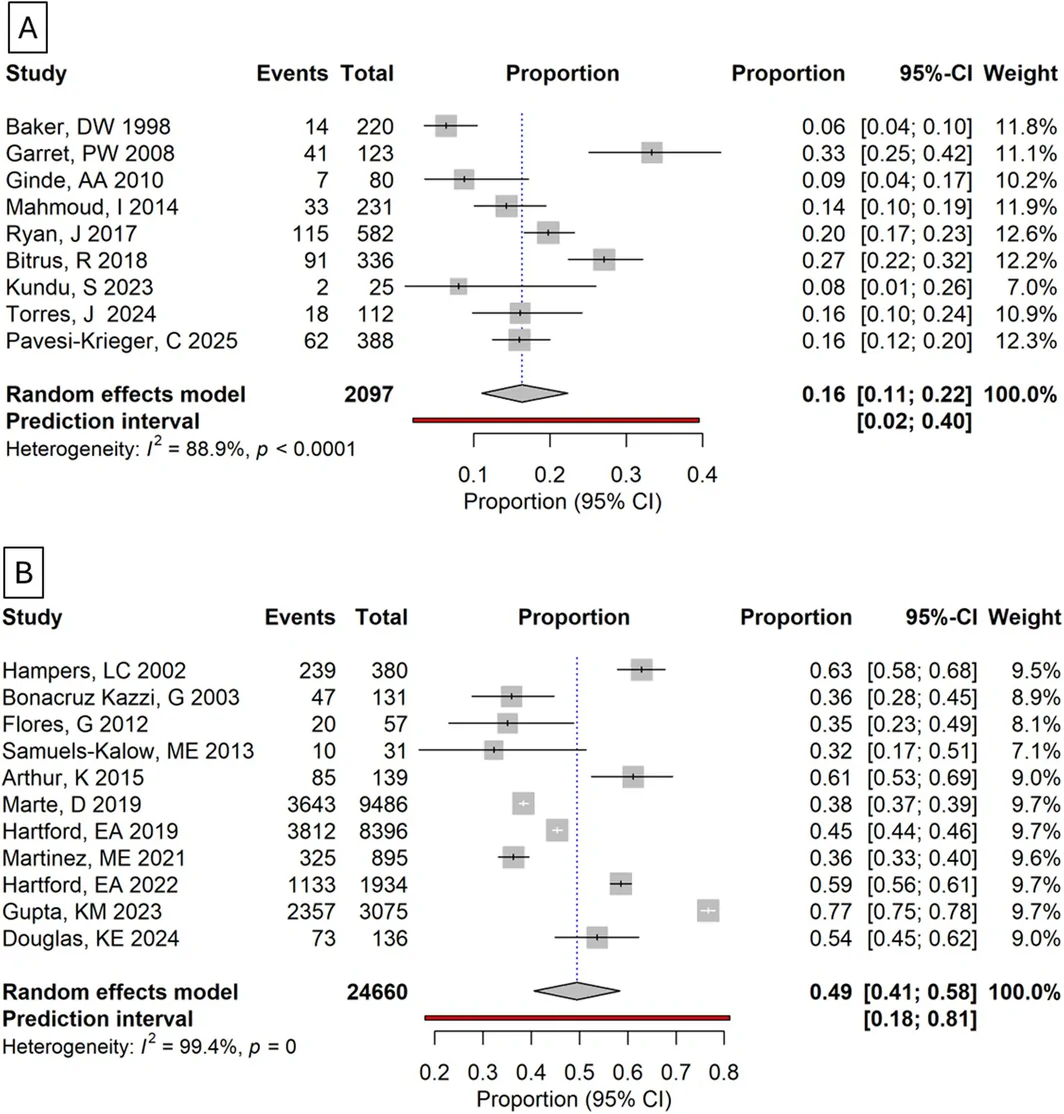
Pooled prevalence of professional interpretation in adults versus children. (A) Studies reporting prevalence for adults. (B) Studies reporting prevalence for children.

**FIGURE 4:**
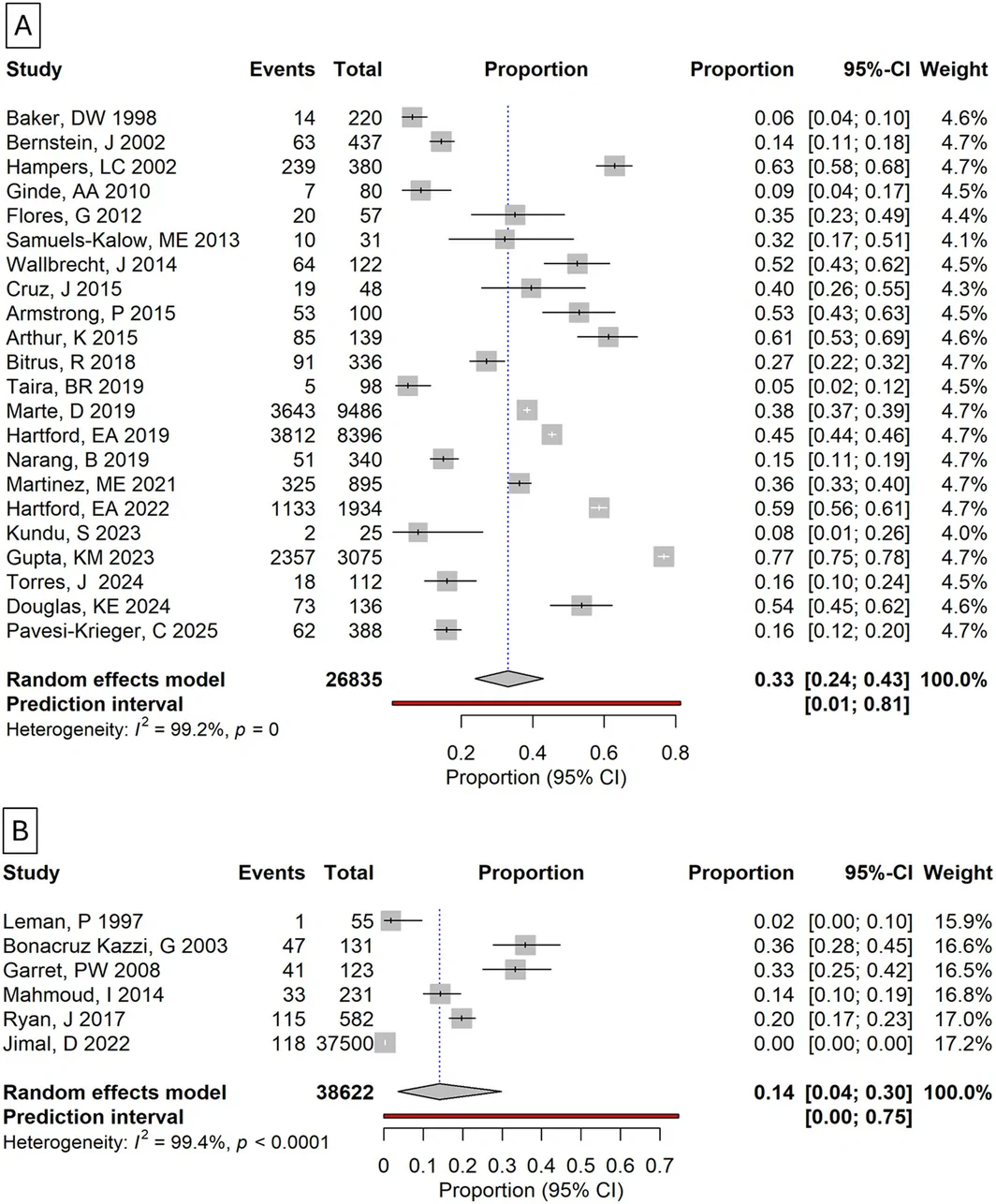
Pooled prevalence of professional interpretation in the United States versus other countries. (A) Studies reporting prevalence in the United States. (B) Studies reporting prevalence outside in other countries.

**FIGURE 5:**
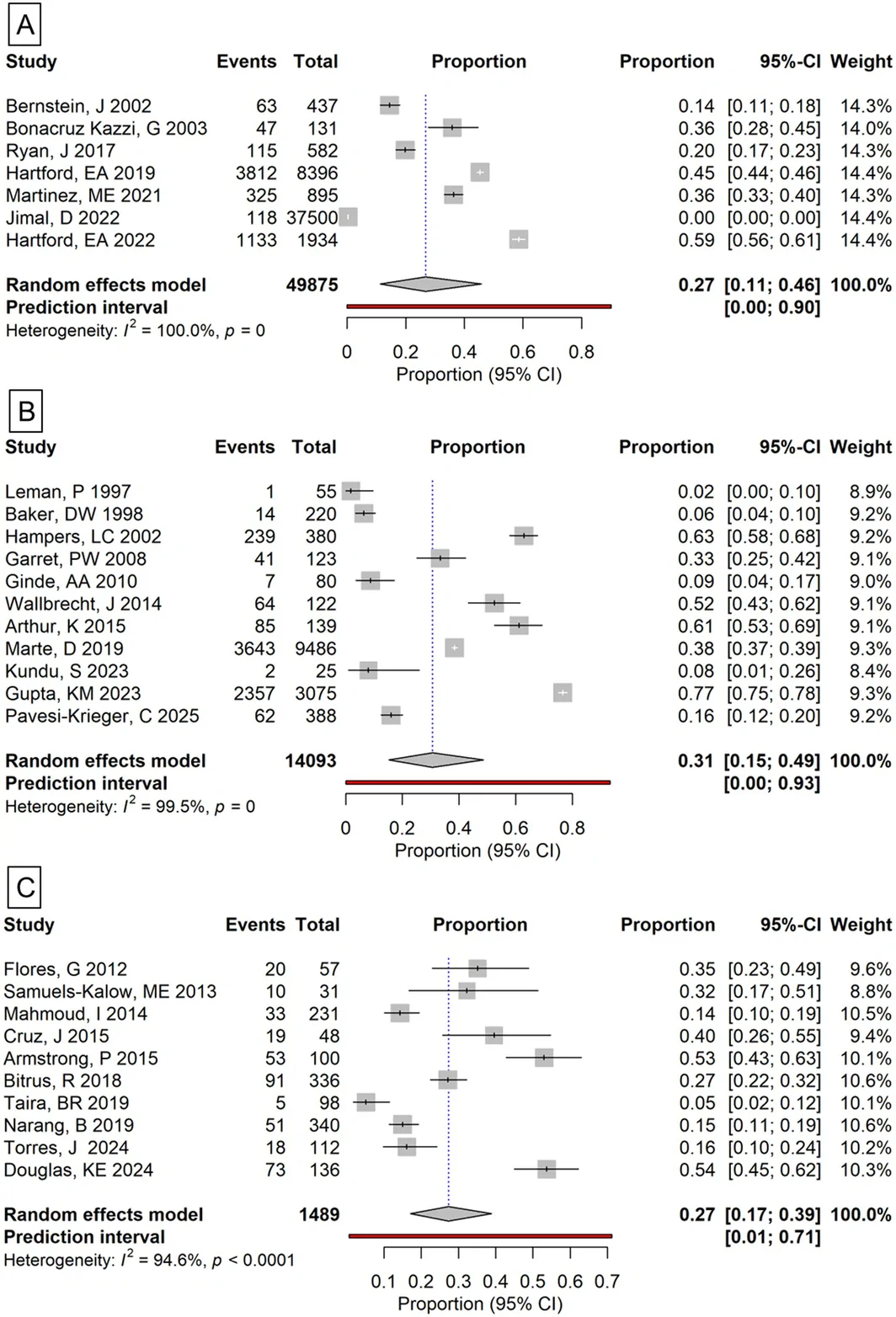
Pooled prevalence of professional interpretation by risk of bias. (A) Studies with low risk of bias. (B) Studies with moderate risk of bias. (C) Studies with high risk of bias.

### 3.4 Meta‐Regression

In univariable random‐effects meta‐regression, study year was not associated with professional interpreter use (*β* = 0.005; 95% CI, −0.006, 0.017; *p* = 0.38). Visual inspection of the main forest plot ordered by publication year (Figure 2) similarly demonstrated no clear temporal trend.

### 3.5 Prevalence by Stage of Care

The four studies not included in the meta‐analysis examined the prevalence of professional interpreter use at specific stages of ED care rather than across the entire visit (Table 3) [33, 43, 44, 58]. These studies reported higher prevalence during physician and nursing assessments compared with triage, registration, reassessment, or disposition.

**TABLE 3:** Summary of studies reporting prevalence by stage of care.

Citation	Country	Population	Setting	Prevalence of professional interpretation by stage					
				Registration	Triage	Nurse assessments	Physician assessments	Reassessments	Disposition
Hartford (2024)	United States	Children (< 18 y/o)	Tertiary pediatric ED			35/49 (71.4%)	45/49 (91.8%)		41/49 (83.7%)
Villalona (2020)	United States	Adult (≥ 18 y/o)	Urban non‐academic ED				54/100 (54.0%)		38/100 (38.0%)
Acharya (2020)	United States	Not specified	ED not specified		76/121 (62.8%)	85/121 (70.2%)	85/121 (70.2%)		
Benda (2019)	United States	Adult (≥ 18 y/o)	Tertiary adult ED	4/7 (57.1%)	4/6 (66.7%)	4/5 (80.0%)	8/10 (80.0%)	12/66 (18.2%)	5/11 (45.5%)

## 4 Discussion

### 4.1 Interpretation of Findings

These results highlight a substantial and ongoing implementation gap in emergency care settings worldwide. Even in settings where professional interpretation services are available, their use remains inconsistent. Variation across populations and countries likely reflects differences in institutional policies, resource availability, interpreter access, and clinician practices. For example, higher interpreter use with children may reflect greater caregiver advocacy, heightened clinician sensitivity to communication barriers for families with children, or institutional policies that prioritize interpretation for pediatric care. The higher prevalence observed in the U.S. may reflect its unique regulatory framework. Under Title VI of the Civil Rights Act of 1964, healthcare organizations receiving federal funding are required to provide language access services, linking compliance to federal financial support [61]. In contrast, countries such as Canada, the United Kingdom, and Australia generally provide guidance on language access but lack comparable enforcement or funding mechanisms. These policy differences may contribute to the observed variation in interpreter use across countries. Critically, our findings suggest that despite well‐established evidence demonstrating the benefits of professional interpretation, the gap in implementation has not narrowed over time.

Between‐study heterogeneity quantified by *I* ^2^ was very high (99%). This is not uncommon in meta‐analyses of prevalence; the PERSyst group analyzed 134 meta‐analyses of prevalence and found a median *I* ^2^ value of 97% [19]. Rather than reflecting methodological limitations alone, this variability likely reflects genuine differences in clinical environments and implementation contexts, given how local availability and awareness impact interpreter uptake [62]. For this reason, prediction intervals, which predict the range of future potential values, were reported to better characterize the range of interpreter use that may occur across EDs.

### 4.2 Comparison to Prior Studies

To our knowledge, this is the first systematic review synthesizing the prevalence of professional interpreter use in healthcare settings. Although previous reviews have outlined the impact of professional interpreters [13, 14], fewer studies have examined how frequently interpretation services are actually used in practice. Existing literature consistently demonstrates that professional interpretation improves communication and outcomes compared to ad hoc or no interpreters [13, 14]. For example, professional interpreters make fewer errors (12% vs. 22%) [51] and result in higher rates of satisfaction (96% vs. 24%) [63], understanding (93% vs. 18%) [63], and defect‐free stroke care (74% vs. 62%) [64]. Yet, qualitative and mixed‐methods studies continue to report barriers to interpreter use, including time pressures, workflow disruption, and limited access to interpretation services [65, 66, 67]. The low prevalence observed in this review highlights a persistent gap between the established benefits of professional interpretation and its routine implementation in emergency departments.

### 4.3 Clinical Implications

Professional interpretation is essential for safe, high‐quality, equitable emergency care, particularly as English‐speaking countries experience growing linguistic diversity. For example, newcomers account for approximately 75% of population growth in Canada [68], underscoring the importance of ensuring equitable communication in healthcare encounters. Our previous scoping review identified numerous modifiable barriers to interpreter use, including limited availability, inadequate technological infrastructure, and lack of familiarity with interpreter services among clinicians and patients [62]. Gutman et al.'s systematic review of implementation strategies identified several approaches that may improve interpreter uptake, including clinician education, increased access to interpreter modalities, standardized institutional policies, and modifications to electronic health records to enable routine identification of patients with language needs [69]. These strategies highlight opportunities for health systems and EDs to improve interpreter use, particularly during reassessments and discharge discussions where communication errors may have important downstream consequences. Establishing accountability mechanisms may further incentivize consistent interpreter use. For example, linking language access requirements to institutional funding (e.g., Title VI of the U.S. Civil Rights Act [61]) or performance metrics (e.g., Joint Commission's National Performance Goal #7 [70]) may encourage more systematic implementation.

### 4.4 Research Implications

Most existing studies have been conducted in academic emergency departments, where interpreter resources and institutional infrastructure may be more readily available. Further research is needed to understand barriers, facilitators, and potential implementation strategies to promote interpreter use in non‐academic and rural settings. The observed differences between children and adults also warrant further investigation, as understanding the drivers of higher interpreter use with children may help identify strategies to improve uptake across all patient populations. While Gutman et al.'s systematic review of implementation strategies found a wide array of effective interventions, heterogeneity in outcomes and limited comparative effectiveness studies made it difficult to determine which strategies were most effective [69]. Evaluating the cost‐effectiveness and resource implications of implementation strategies will be particularly important, given that financial constraints remain a major barrier to interpreter access. Lastly, pre‐clinical studies have tested the use of artificial intelligence (AI), such as neural machine translation (e.g., Google Translate) and large language models (e.g., ChatGPT), for the translation of written materials [71, 72, 73]. However, these tools have not yet been implemented for patient care nor tested for real‐time interpretation (i.e., spoken word), likely in part due to their poor performance for low‐resource languages [71, 72, 73]. This remains an area of future research.

### 4.5 Limitations

This review has certain limitations. First, we limited inclusion to English‐speaking ED settings and databases primarily indexing English‐language literature, which may limit the generalizability of findings to other healthcare systems. While we included 35 non‐English languages in the search strategy, we recognize that this was not exhaustive and therefore we could have missed studies involving other languages. However, given our focus on English‐speaking settings and interpretation or translation between English and a non‐English language, we anticipate that any missed studies would not have impacted our findings in a meaningful way. Our analysis focused specifically on professional interpreter use and we excluded encounters with bilingual clinicians or where patients declined interpretation from the denominator. However, this relied on individual study investigators documenting these situations, which was not common. This may result in an under‐estimate of the prevalence relative to the true value, as the denominator of patients with language needs may be smaller than currently calculated. Furthermore, because we extracted only pre‐intervention time points from before–after or interrupted time series studies, our estimates may underestimate interpreter use in settings where implementation interventions have subsequently improved access. Finally, substantial heterogeneity across studies limits the interpretability of a single pooled prevalence estimate. However, such variation is expected when synthesizing healthcare practices across diverse ED environments, and the use of prediction intervals helps contextualize the range of interpreter use that may occur across settings [19].

## 5 Conclusion

In conclusion, professional interpreter use in English‐speaking EDs remains low, with fewer than one‐third of patients with preferred languages other than English receiving professional interpretation. Use appears higher among pediatric encounters and in the U.S. compared with other countries, suggesting that institutional, population, and policy factors influence implementation. Despite well‐established benefits of professional interpretation, our findings demonstrate a persistent gap between evidence and practice. Addressing this gap will require coordinated efforts from clinicians, health system leaders, and policymakers to improve access to and accountability for language‐concordant care in emergency settings.

## Author Contributions

H.L. conceptualized the study. H.L., L.H., J.K., L.D., V.D., K.G., and S.A. contributed to study design. H.L. and A.R. contributed to screening and data extraction. H.L. led data analysis. All authors contributed to the results interpretation. H.L. and A.R. drafted the manuscript. All authors critically revised the manuscript for important intellectual content and approved the final version. H.L. takes responsibility for the paper as a whole.

## Funding

A.R. was funded by an Alberta Innovates Summer Studentship (2025) to work on this project. L.H. is supported by a Canada Research Chair in Knowledge Synthesis and Translation.

## Conflicts of Interest

The authors declare no conflicts of interest.

## Supporting information


**Figure S1:** Funnel plot of included studies.

**Appendix 2.** Search Strategy.

**Table S1:** PRISMA Checklist.

**Table S2:** Eligibility criteria of preceding scoping review.

**Table S3:** Data Extraction Form.

**Table S4:** Characteristics of individual studies.

## Data Availability

The data that support the findings of this study are available from the corresponding author upon reasonable request.
