# Supplementary material for: Prevalence of Professional Interpreter Use in Emergency Departments: A Systematic Review and Meta‐Analysis
Source: Acad Emerg Med. 2026 Oct 14;33(10):e70427. doi: 10.1111/acem.70427 (PMC13664663; doi:10.1111/acem.70427)
Supplement: Supplementary file 1 [file ACEM-33-0-s002.docx]

**Appendix 2. Search Strategy**

**Ovid MEDLINE(R) ALL <1946 to May 19, 2025>**

Date searched: May 20, 2025

Results: 731

1	Translating/	6461

2	Translations/	7840

3	(translator* or interpreter* or (translat* and (refugee* or migrant worker* or immigrant* or newcomer* or english proficiency or "english as a second language" or "non-native speaker*" or foreign language* or "non-english speak*")) or (translat* adj6 (speak* or language* or speech or conversation* or bilingual or multilingual or multi-lingual or google or app or apps or application* or smartphone* or "smart phone*" or "cell phone*" or "mobile phone*" or "mobile device*" or tablet or tablets or ipad* or english or spanish or hispanic* or french or german or chinese or japanese or tagalog or portugese or mandarin or cantonese or hindi or russian or ukrainian or arabic or urdu or bengali or indonesian or persian or farsi or dutch or polish or punjabi or korean or romanian or cree or hungarian or greek or italian or tamil or vietnamese or serbian or croatian or somali or gujarati or hindustani)) or (translat* adj3 (tool or tools or service or services))).mp.	39265

4	1 or 2 or 3	46306

5	Emergency Treatment/ or Emergency Medicine/ or emergency medical services/ or emergency room visits/ or exp emergency service, hospital/ or emergency services, psychiatric/ or trauma centers/ or triage/ or exp Evidence-Based Emergency Medicine/ or exp Emergency Nursing/ or Emergencies/ or (emergicent* or casualty department* or triage or ((emergenc* or ED) adj2 (room* or accident or ward or wards or unit or units or department* or physician* or doctor* or nurs* or treatment* or presentation or visit or visits or setting or patient or patients or medicine or care)) or (trauma adj1 (cent* or care))).mp.	392090

6	4 and 5	740

7	limit 6 to (comment or editorial or letter or news or newspaper article)	9

8	6 not 7	731

**Embase <1974 to 2025 May 19> (OVID Interface)**

Date searched: May 20, 2025

Results: 1312

1	"translating (language)"/	3219

2	(translator* or interpreter* or (translat* and (refugee* or migrant worker* or immigrant* or newcomer* or english proficiency or "english as a second language" or "non-native speaker*" or foreign language* or "non-english speak*")) or (translat* adj6 (speak* or language* or speech or conversation* or bilingual or multilingual or multi-lingual or google or app or apps or application* or smartphone* or "smart phone*" or "cell phone*" or "mobile phone*" or "mobile device*" or tablet or tablets or ipad* or english or spanish or hispanic* or french or german or chinese or japanese or tagalog or portugese or mandarin or cantonese or hindi or russian or ukrainian or arabic or urdu or bengali or indonesian or persian or farsi or dutch or polish or punjabi or korean or romanian or cree or hungarian or greek or italian or tamil or vietnamese or serbian or croatian or somali or gujarati or hindustani)) or (translat* adj3 (tool or tools or service or services))).mp.	52218

3	1 or 2	52218

4	emergency treatment/ or emergency medicine/ or emergency health service/ or emergency outpatient clinic/ or hospital emergency service/ or patient triage/ or psychiatric emergency service/ or evidence based emergency medicine/ or emergency nursing/ or emergency care/ or emergency ward/ or emergency/ or (emergicent* or "casualty department*" or triage* or ((emergenc* or ED) adj2 (room* or accident or ward or wards or unit or units or department* or physician* or doctor* or nurs* or treatment* or presentation or visit or visits or setting or patient or patients or medicine or care)) or (trauma adj1 (cent* or care))).mp.	663065

5	3 and 4	1326

6	limit 5 to (editorial or letter)	14

7	5 not 6	1312

**APA PsycInfo <1806 to May 2025 Week 2> (OVID Interface)**

Date searched: May 20, 2025

Results: 155

1	exp foreign language translation/	19518

2	interpreters/	1162

3	(translator* or interpreter* or (translat* and (refugee* or migrant worker* or immigrant* or newcomer* or english proficiency or "english as a second language" or "non-native speaker*" or foreign language* or "non-english speak*")) or (translat* adj6 (speak* or language* or speech or conversation* or bilingual or multilingual or multi-lingual or google or app or apps or application* or smartphone* or "smart phone*" or "cell phone*" or "mobile phone*" or "mobile device*" or tablet or tablets or ipad* or english or spanish or hispanic* or french or german or chinese or japanese or tagalog or portugese or mandarin or cantonese or hindi or russian or ukrainian or arabic or urdu or bengali or indonesian or persian or farsi or dutch or polish or punjabi or korean or romanian or cree or hungarian or greek or italian or tamil or vietnamese or serbian or croatian or somali or gujarati or hindustani)) or (translat* adj3 (tool or tools or service or services))).mp.	37393

4	1 or 2 or 3	37409

5	Emergency medicine/ or (emergicent* or triage or "casualty department*" or ((emergenc* or ed) adj2 (room* or accident or ward or wards or unit or units or department* or physician* or doctor* or nurs* or treatment* or presentation or visit or visits or setting or patient or patients or medicine or care)) or (trauma adj1 (cent* or care))).mp.	27089

6	4 and 5	157

7	limit 6 to ("column/opinion" or editorial or letter)	2

8	6 not 7	155

**CINAHL Plus with Full Text (EBSCOhost interface)**

Date searched: May 20, 2025

Results: 576

S1 (MH "Medical Interpretation Services") OR (MH "Medical Interpreters") OR (MH "Translations") OR translator* or interpreter* or (translat* and (refugee* or "migrant worker*" or immigrant* or newcomer* or "english proficiency" or "english as a second language" or "non-native speaker*" or "foreign language*" or "non-english speak*")) or (translat* N6 (speak* or language* or speech or conversation* or bilingual or multilingual or multi-lingual or google or app or apps or application* or smartphone* or "smart phone*" or "cell phone*" or "mobile phone*" or "mobile device*" or tablet or tablets or ipad* or english or spanish or hispanic* or french or german or chinese or japanese or tagalog or portugese or mandarin or cantonese or hindi or russian or ukrainian or arabic or urdu or bengali or indonesian or persian or farsi or dutch or polish or punjabi or korean or romanian or cree or hungarian or greek or italian or tamil or vietnamese or serbian or croatian or somali or gujarati or hindustani)) or (translat* N3 (tool or tools or service or services))

S2 (MH "Emergency Service+") or (MH "Emergency Medicine") or (MH "Physicians, Emergency") OR (MH "Emergency Nurse Practitioners") or (MH "Emergency Nursing+") or (MH "Emergency Patients") or emergicent* or "casualty department*" or triage or ((emergenc* or "ED") N2 (room* or accident or ward or wards or unit or units or department* or physician* or doctor* or nurs* or treatment* or presentation OR visit OR visits OR setting OR patient OR patients OR medicine OR care)) or (trauma N1 (cent* or care))

S3 S1 AND S2

S4 S1 AND S2	**Limiters** - Publication Type: Commentary, Editorial, Letter

S5 S3 NOT S4

**Scopus (Advanced search)**

Date searched: May 20, 2025

Results: 950

( TITLE-ABS-KEY ( translator* OR interpreter* OR ( translat* AND ( refugee* OR "migrant worker*" OR immigrant* OR newcomer* OR "english proficiency" OR "english as a second language" OR "non-native speaker*" OR "foreign language*" OR "non-english speak*" ) ) OR ( translat* W/6 ( speak* OR language* OR speech OR conversation* OR bilingual OR multilingual OR multi-lingual OR google OR app OR apps OR application* OR smartphone* OR "smart phone*" OR "cell phone*" OR "mobile phone*" OR "mobile device*" OR tablet OR tablets OR ipad* OR english OR spanish OR hispanic* OR french OR german OR chinese OR japanese OR tagalog OR portugese OR mandarin OR cantonese OR hindi OR russian OR ukrainian OR arabic OR urdu OR bengali OR indonesian OR persian OR farsi OR dutch OR polish OR punjabi OR korean OR romanian OR cree OR hungarian OR greek OR italian OR tamil OR vietnamese OR serbian OR croatian OR somali OR gujarati OR hindustani ) ) OR ( translat* W/3 ( tool OR tools OR service OR services ) ) ) AND TITLE-ABS-KEY ( emergicent* OR "casualty department*" OR triage OR ( ( emergenc* OR "ED" ) W/2 ( room* OR accident OR ward OR wards OR unit OR units OR department* OR physician* OR doctor* OR nurs* OR treatment* OR presentation OR visit OR visits OR setting OR patient OR patients OR medicine OR care ) ) OR ( trauma W/1 ( cent* OR care ) ) ) ) AND ( EXCLUDE ( DOCTYPE , "le" ) OR EXCLUDE ( DOCTYPE , "ed" ) )

**Cochrane Library - CENTRAL trials database only (Wiley interface)**
Date searched: May 20, 2025

Results: 99

#1 [mh ^"translating"] or [mh ^"translations"] or (translator* or interpreter* or (translat* and (refugee* or migrant-workers or immigrant* or newcomer* or "english proficiency" or "english as a second language" or non-native-speaker or foreign-language or (non-english NEXT speak*))) or (translat* NEAR/6 (speak* or language* or speech or conversation* or bilingual or multilingual or multi-lingual or google or app or apps or application* or smartphone* or "smart phone" or "cell phone" or "mobile phone" or "mobile device" or tablet or tablets or ipad* or english or spanish or hispanic* or french or german or chinese or japanese or tagalog or portugese or mandarin or cantonese or hindi or russian or ukrainian or arabic or urdu or bengali or indonesian or persian or farsi or dutch or polish or punjabi or korean or romanian or cree or hungarian or greek or italian or tamil or vietnamese or serbian or croatian or somali or gujarati or hindustani)) or (translat* NEAR/3 (tool or tools or service or services))):ti,ab,kw

#2 [mh ^"emergency treatment"] or [mh ^"emergency medicine"] or [mh ^"emergency medical services"] or [mh "emergency room visits"] or [mh ^"emergency service, hospital"] or [mh ^"emergency services, psychiatric"] or [mh ^"trauma centers"] or [mh ^"triage"] or [mh "evidence based emergency medicine"] or [mh "emergency nursing"] or [mh ^"emergencies"] or (triage or emericent* or "casualty department" or "A&E" or "A and E" or (trauma NEAR/1 (cent* or care))):ti,ab,kw or ((emergenc* or ED) NEAR/2 (room* or accident or ward or wards or unit or units or department* or physician* or doctor* or nurs* or treatment* or presentation OR visit OR visits OR setting OR patient OR patients OR medicine OR care)):ti,ab,kw

#3	#1 AND #2

**iPortal <https://iportal.usask.ca/>**

Date searched: May 20, 2025 (no new for update)

"Emergency room" 4 results

"Emergency department" 24 results

"Emergency care"	4 results

"Emergency ward"	2 results

**Native health database (**[**https://nativehealthdatabase.net/**](https://nativehealthdatabase.net/)**)**

**Date searched: May 20, 2025** (no new for update)

Translating (7 results)

Translation (23 results)

Translators (0 results)

Interpreters (8 results - but 1 duplicate)
