# Supplementary material for: Prevalence of Professional Interpreter Use in Emergency Departments: A Systematic Review and Meta‐Analysis
Source: Acad Emerg Med. 2026 Oct 14;33(10):e70427. doi: 10.1111/acem.70427 (PMC13664663; doi:10.1111/acem.70427)
Supplement: Supplementary file 2 [file ACEM-33-0-s003.docx]

**Table S3. Data Extraction Form**

| **Study characteristics** | |
| --- | --- |
| Study ID | Free text |
| DOI | Free text |
| Full text or Abstract | Full text, abstract |
| First author | Free text |
| Publication year | Free text |
| Country where study was conducted | Free text |
| Study design | Cross-sectional, prospective cohort, retrospective cohort, before-after, interrupted time series (without comparison), case-control, nested case control, non-concurrent cohort, controlled before-after, interrupted time series (with comparison), other (please specify) |
| **Setting characteristics** | |
| Number of sites | Free text |
| ED names | Free text |
| City names | Free text |
| Setting (select all) | General ED, adult ED, pediatric ED, urgent care, other (specify) |
| Type of ED (select all) | Tertiary academic, urban non-academic, regional, rural, other (specify) |
| Top 5 non-English languages for each city | Free text |
| City census (population) | Free text |
| Private or public ownership | Private, public, unclear, other |
| ED volume (patients seen per year) | Free text |
| Types of professional interpretation modalities available (select all) | In-person, audiovisual. telephonic, other (please specify) |
| In-person interpreters in house | Yes, No, Unclear, N/A |
| Dedicated IT support line for audiovisual/telephonic interpretation | Yes, No, Unclear, N/A |
| Interpretation policy present | Yes, No, Unclear |
| Training provided for healthcare workers | Yes, No, Unclear |
| Reminders in ED present | Yes, No, Unclear |
| **EMR characteristics** | |
| Presence of EMR | Yes, No, Unclear |
| EMR vendor | Epic, Cerner, Meditech, Other (please specify) |
| Reminders in EMR present | Yes, No, Unclear |
| Structured field in EMR for language | Yes, No, Unclear |
| **Sample characteristics** | |
| Inclusion criteria for study | Free text |
| Exclusion criteria for study | Free text |
| Other information about sample | Free text |
| Total number of LEP patients | Drop-down/numeric |
| Patient ages | Adult (>=18yo), Children (<18yo), Both |
| Languages spoken | Free text |
| **Prevalence of interpretation for all patients (N or % if not available)** | |
| All professional modalities | Free text |
| Professional in-person | Free text |
| Professional audiovisual | Free text |
| Professional telephonic | Free text |
| Other (please specify) | Free text |
| **Prevalence of interpretation for adult patients (N or % if not available)** | |
| All professional modalities | Free text |
| Professional in-person | Free text |
| Professional audiovisual | Free text |
| Professional telephonic | Free text |
| Other (please specify) | Free text |
| **Prevalence of interpretation for pediatric patients (N or % if not available)** | |
| All professional modalities | Free text |
| Professional in-person | Free text |
| Professional audiovisual | Free text |
| Professional telephonic | Free text |
| Other (please specify) | Free text |
| **Quality assessment** | |
| Was the sample frame appropriate to address the target population? | Yes, No, Unclear |
| Were study participants sampled in an appropriate way? | Yes, No, Unclear |
| Was the sample size adequate? | Yes, No, Unclear |
| Were the study subjects and the setting described in detail? | Yes, No, Unclear |
| Was the data analysis conducted with sufficient coverage of the identified sample? | Yes, No, Unclear |
| Were valid methods used for the identification of the condition? | Yes, No, Unclear |
| Was the condition measured in a standard, reliable way for all participants? | Yes, No, Unclear |
| Was there appropriate statistical analysis? | Yes, No, Unclear |
| Was the response rate adequate, and if not, was the low response rate managed appropriately? | Yes, No, Unclear |
