# Supplementary material for: Prevalence of Professional Interpreter Use in Emergency Departments: A Systematic Review and Meta‐Analysis
Source: Acad Emerg Med. 2026 Oct 14;33(10):e70427. doi: 10.1111/acem.70427 (PMC13664663; doi:10.1111/acem.70427)
Supplement: Supplementary file 3 [file ACEM-33-0-s004.docx]

**Table S4. Characteristics of individual studies**

| Study ID | Full text or Abstract | Publication Year | Country | Study design | # sites | Setting | Modality | # patients | Patient ages | Languages spoken |
| --- | --- | --- | --- | --- | --- | --- | --- | --- | --- | --- |
| Leman 1997 | Full text | 1997 | United Kingdom | Cross-sectional | 1 | General ED | Not specified | 55 | Both | 33 different languages |
| Baker 1998 | Full text | 1998 | United States | Cross-sectional | 1 | General ED | Not specified | 220 | Adult (>= 18 y/o) | Spanish |
| Bernstein 2002 | Full text | 2002 | United States | Retrospective cohort | 1 | ED not specified | In-person | 437 | Not specified | Spanish, Portuguese Creole, Haitian Creole |
| Hampers 2002 | Full text | 2002 | United States | Prospective cohort | 1 | Pediatric ED | In-person | 380 | Children (<18 y/o) | Spanish, Polish, Russian, Vietnamese |
| BonacruzKazzi 2003 | Full text | 2003 | Australia | Cross-sectional | 1 | General ED | In-person ; Telephonic | 131 | Children (<18 y/o) | 22 different languages |
| Garrett 2008 | Full text | 2008 | Australia | Retrospective cohort | 1 | General ED | Not specified | 123 | Adult (>= 18 y/o) | 9 different languages |
| Ginde 2010 | Full text | 2010 | United States | Cross-sectional | 4 | ED not specified | Not specified | 80 | Adult (>= 18 y/o) | Spanish, others |
| Flores 2012 | Full text | 2012 | United States | Prospective cohort | 2 | Pediatric ED | Not specified | 57 | Children (<18 y/o) | Spanish |
| Samuels-Kalow 2013 | Full text | 2013 | United States | Prospective cohort | 1 | Pediatric ED | In-person ; Telephonic | 31 | Children (<18 y/o) | Spanish |
| Mahmoud 2014 | Full text | 2014 | Australia | Cross-sectional | 1 | Adult ED | Not specified | 231 | Adult (>= 18 y/o) | Not specified |
| Wallbrecht 2014 | Full text | 2014 | United States | Prospective cohort | 1 | General ED | Audiovisual; Telephonic | 122 | Both | Spanish, Navajo, Vietnamese, Chinese, Arabic, ASL |
| Armstrong 2015 | Abstract | 2015 | United States | Prospective cohort | 1 | ED not specified | Telephonic | 100 | Not specified | Spanish, ASL, Amharic, Arabic, Mandarin |
| Arthur 2015 | Full text | 2015 | United States | Cross-sectional | 1 | Pediatric ED | In-person ; Telephonic | 139 | Children (<18 y/o) | Spanish |
| Cruz 2015 | Abstract | 2015 | United States | Cross-sectional | 1 | ED not specified | Not specified | 48 | Not specified | Spanish |
| Ryan 2017 | Full text | 2017 | Australia | Retrospective cohort | 1 | Adult ED | In-person ; Telephonic | 582 | Adult (>= 18 y/o) | 10 different languages |
| Bitrus 2018 | Abstract | 2018 | United States | Cross-sectional | 1 | ED not specified | Not specified | 336 | Adult (>= 18 y/o) | Spanish, Arabic |
| Benda 2019 | Full text | 2019 | United States | Prospective cohort | 1 | Adult ED | In-person ; Telephonic | 9 | Adult (>= 18 y/o) | Spanish |
| Hartford 2019 | Full text | 2019 | United States | Retrospective cohort | 1 | Pediatric ED | In-person ; Audiovisual; Telephonic | 8396 | Children (<18 y/o) | 10 different languages |
| Marte 2019 | Abstract | 2019 | United States | Retrospective cohort | 1 | Pediatric ED | Not specified | 9486 | Children (<18 y/o) | Spanish |
| Narang 2019 | Full text | 2019 | United States | Before-after (without comparison) | 1 | Urgent care | Telephonic | 340 | Not specified | 13 different languages |
| Taira 2019 | Full text | 2019 | United States | Retrospective cohort | 1 | ED not specified | Audiovisual; Telephonic | 98 | Not specified | Spanish, Armenian, Tagalog |
| Acharya 2020 | Abstract | 2020 | United States | Cross-sectional | 1 | ED not specified | Not specified | 121 | Not specified | Spanish, Others |
| Villalona 2020 | Full text | 2020 | United States | Cross-sectional | 1 | ED not specified | Audiovisual; Telephonic | 100 | Adult (>= 18 y/o) | Spanish |
| Martinez 2021 | Full text | 2021 | United States | Interrupted time series (with comparison) | 1 | Pediatric ED; Urgent care | In-person ; Audiovisual; Telephonic; Certified HCW | 895 | Children (<18 y/o) | Spanish |
| Hartford 2022 | Full text | 2022 | United States | Interrupted time series (without comparison) | 1 | Pediatric ED | In-person ; Audiovisual; Telephonic | 1934 | Children (<18 y/o) | 9 different languages |
| Jimal 2022 | Full text | 2022 | Canada | Retrospective cohort | 1 | General ED | Telephonic | 37500 | Both | Arabic, French, Mandarin, Korean, Spanish, Somali, Others |
| Gupta 2023 | Full text | 2023 | United States | Interrupted time series (without comparison) | 1 | Pediatric ED | In-person ; Audiovisual; Telephonic | 3075 | Children (<18 y/o) | Spanish, Arabic, Mandarin, Portuguese, Bengali, and Vietnamese |
| Kundu 2023 | Full text | 2023 | United States | Retrospective cohort | 1 | ED not specified | In-person ; Audiovisual; Telephonic | 25 | Adult (>= 18 y/o) | Spanish |
| Douglas 2024 | Full text | 2024 | United States | Interrupted time series (without comparison) | 1 | Pediatric ED | In-person ; Audiovisual; Telephonic | 136 | Children (<18 y/o) | Many |
| Hartford 2024 | Full text | 2024 | United States | Cross-sectional | 1 | Pediatric ED | Not specified | 49 | Children (<18 y/o) | 15 different languages |
| Torres 2024 | Abstract | 2024 | United States | Cross-sectional | 1 | ED not specified | Telephonic | 112 | Adult (>= 18 y/o) | Spanish |
| Pavesi-Krieger 2025 | Full text | 2025 | United States | Retrospective cohort | 1 | ED not specified | In-person ; Audiovisual; Telephonic; Certified HCW | 388 | Adult (>= 18 y/o) | Not specified |
