# Supplementary material for: Prevalence of Professional Interpreter Use in Emergency Departments: A Systematic Review and Meta‐Analysis
Source: Acad Emerg Med. 2026 Oct 14;33(10):e70427. doi: 10.1111/acem.70427 (PMC13664663; doi:10.1111/acem.70427)
Supplement: Supplementary file 4 [file ACEM-33-0-s005.docx]

**Table S2. Eligibility criteria of preceding scoping review**

| Characteristic | Inclusion Criteria | Exclusion Criteria |
| --- | --- | --- |
| Population | Patients of all ages (pediatric and adult) with a preferred language other than English  Note: the determination of preferred language may be based on various factors and it is important to consider the diverse ways it may be identified across studies. These include, but are not limited to, the individual's primary language spoken at home or self-reported preferred language | Persons whose preferred language is English |
| Concept | Translation/interpretation of text, audio, or spoken/signed language between English and a non-English language during an emergency patient encounter  Note: Translation/interpretation may occur bidirectionally or unidirectionally. Studies may involve the implementation of an interpretation/translation modality, evaluation of an implemented interpretation/translation modality, or both. | Translation/interpretation between two non-English languages or that occurs outside of an emergency patient encounter (e.g. follow-up after discharge, translation of general resources, translation of documents unrelated to direct patient care) |
| Context | Emergency department (General, Adult, OR Pediatric), urgent care centre, or pre-hospital setting where English is the primary working language | Physician offices, in- or outpatient facilities, phlebotomy labs, dental clinics, inpatient hospital wards, long-term care homes, standardized patients/simulation/non-clinical |
| Publication Date | Inception to current | None |
| Study Design | Descriptive, observational, quantitative, qualitative, mixed methods | Editorials, letters, opinion pieces, policy recommendations, case reports, and case series |
| Publication Status | Published in a peer-reviewed journal, abstracts, conference proceedings and grey literature | Non-peer-reviewed articles, personal blogs/websites, textbooks or book chapters |
| Language | Any | None |
| Country | Any | None |
